# Genotype-phenotype: correlations and emerging spectrum

**DOI:** 10.1186/1471-2474-14-S2-O3

**Published:** 2013-05-29

**Authors:** Arnold Reuser

**Affiliations:** 1 Department of Clinical Genetics, Center for Lysosomal and Metabolic Diseases, Erasmus MC University Medical Center, Rotterdam, The Netherlands

## 

The Pompe disease mutation database at http://www.pompecenter.nl is a handy tool to quickly learn about the effect of sequence variations in the *GAA* gene. The database aims to be complete and to contain all the *GAA* sequence variations that have been published. The information is regularly updated and electronically linked to relevant publications. Nevertheless, there is a shortcoming in that the database only indirectly links genotypes to phenotypes via the linked publications. This problem has, in part, been solved by inserting a column for reporting the severity of individual mutations. The ‘severity score’ of each sequence variant is partly based on the nature of the mutation. For instance, most frame shifts in the *GAA* gene are bound to cause full loss of functional enzyme. Missense mutations, however, have an unpredictable effect, but can be investigated in a transient expression system. In such case, a sophisticated scoring system provides the severity score in terms of ‘very severe,’ ‘potentially less severe,’ ‘potentially mild’ etc. A similar grading system for splice site mutations is not yet in place. Ideally, the database should also provide prognostic information on patients’ clinical outcomes based on genotype-phenotype correlations, if such correlations, though expected, in fact exist. The phenotype could then be predicted from the composite genotype. For instance, if both *GAA* mutations of the patient were listed as ‘very severe,’ the patient would predictably develop classic infantile Pompe disease, but would predictably develop an attenuated phenotype when having one ‘severe’ and one ‘mild’ mutation. A preliminary and incomplete analysis of the data contained in the Pompe disease mutation database suggests that many mutations can be classified in terms of ‘classic infantile’ mutations, typically ‘childhood’ mutations, or typically ‘adult’ mutations. The most common mutation of all i.e. c.-32-13T>G (IVS1) is difficult to classify. In combination with any ‘classic infantile’ mutation, it correlates with onset of symptoms before the first year of life till onset in late adulthood, but it is never associated with a very rapid demise. In these cases, the genotype-phenotype correlation demonstrates the impact of modifying factors. This topic will be discussed with regard to diagnosis, prognosis, and treatment.

